# Protocol for isolation of total RNA from mouse whole cochlea using organic solvents

**DOI:** 10.1016/j.xpro.2026.104376

**Published:** 2026-02-16

**Authors:** Ezequiel Rías, Guillermo Spitzmaul, Leonardo Dionisio

**Affiliations:** 1 Instituto de Investigaciones Bioquímicas de Bahía Blanca, CONICET-UNS, Bahía Blanca 8000, Argentina; 2 Departamento de Biología, Bioquímica y Farmacia, Universidad Nacional del Sur (UNS), Bahía Blanca 8000, Argentina

**Keywords:** Genetics, Model Organisms, Gene Expression, Systems biology

## Abstract

Determining gene expression changes in cochlear components is important for understanding the mechanisms underlying hearing loss. Here, we present a protocol for the isolation of total RNA from mouse cochleae across a wide range of ages (2–25 weeks old) using organic solvent extraction. We describe the steps for obtaining the entire cochlea from mice and isolating high-quality total RNA suitable for further reverse transcription and PCR.

For complete details on the use and execution of this protocol, please refer to Rías et al.^1^

## Highlights

• Protocol for RNA isolation from mouse whole cochlea
• Procedure for cochlea homogenization
• Steps for aqueous phase obtention and RNA precipitation
• Guidance for RNA quality testing and quantification

## Abstract (editor highlights)

Publisher’s note: Undertaking any experimental protocol requires adherence to local institutional guidelines for laboratory safety and ethics.

## Graphical abstract

**Figure:**
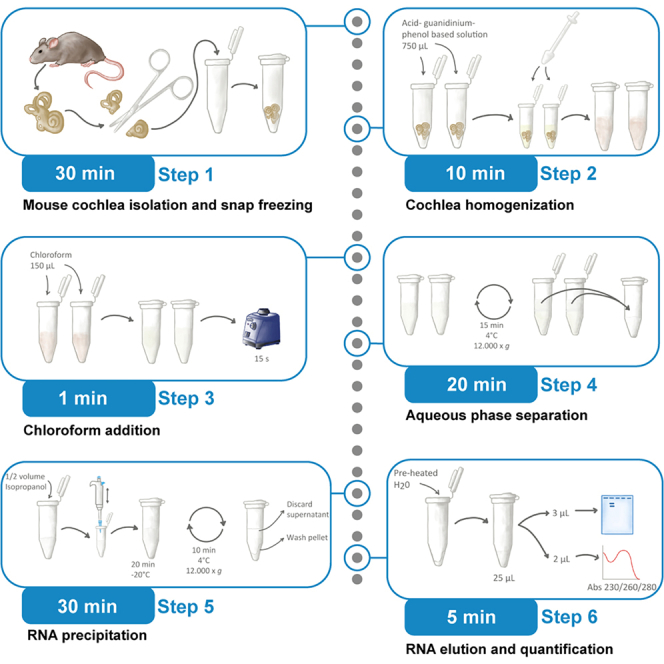

## Before you begin

This protocol is adapted from the classical organic solvent-based RNA extraction method described by Chomczynski and Sacchi (1987).^2^ It has been optimized to obtain high-quality total RNA from mice cochleae, with yields and purity suitable for downstream molecular analyses such as reverse transcription and quantitative PCR. We have successfully used this procedure to assess gene expression in different cochlear components under both normal and pathological conditions (Rías et al., 2025).^1^

The day before to cochleae dissection:

1. Store 1X PBS at 4°C.

***Note:*** A homemade 1X PBS solution prepared with ultrapure water can be used.

2. Place a cooling device to use as a base for cochlear dissection in the freezer. A frozen petri dish of 7 cm diameter could be used for this purpose.
3. Prepare 70% ethanol using ultrapure water (PB-L Productos Bio-Lógicos, cat N° IA0602) and high-quality absolute ethanol (J. T. Baker). Store at −20°C.

### Innovation

This protocol describes an optimized adaptation of the classical RNA extraction method by Chomczynski and Sacchi (1987),^2^ specifically designed for the mouse cochlea—a small, calcified, and RNA-sensitive tissue where standard procedures often result in low yield or degradation. The workflow has been refined to improve tissue handling, homogenization, and phase separation, preserving RNA integrity and maximizing recovery. Key improvements include efficient otic capsule disruption, adjusted reagent volumes, and a standardized sample pooling strategy in which two tubes, each containing three cochleae (three cochleae from three mice) are processed in parallel to ensure consistency. Using only common laboratory reagents and equipment, this protocol provides high-quality RNA suitable for reverse transcription and quantitative PCR. Overall, it offers a cost-effective, reproducible alternative to commercial kits, facilitating reliable gene expression studies in wild-type and transgenic mouse cochleae, and expanding molecular research possibilities in auditory biology.

### Institutional permissions

All experiments described here were conducted in accordance with the animal care regulations approved by CICUAE N° 253-2024, UNS. In brief, animals were housed in the BSA-Bioterio UNS-CONICET accredited animal facility with controlled temperature (25°C), humidity, and lighting (alternating 12 h light/dark cycles). Food and water were provided *ad libitum.* The *Kcnq4*^−/−^ mice used in this study are a genetic model of hearing loss and were generated lacking the expression of the potassium channel KCNQ4.^1^^,^^3^^,^^4^ Wild type (WT) and *Kcnq4*^−/−^ mice on a C3H/HeJ genetic background were used at 4 post-natal weeks (W). This protocol, however, can be applied to cochleae from mice of different ages and different genetic backgrounds. Both male and female mice were used without distinction.

## Key resources table

**Table:** 

REAGENT or RESOURCE	SOURCE	IDENTIFIER
**Chemicals, peptides, and recombinant proteins**		

BIO-ZOL	PB-L Productos Bio-Lógicos	cat N° RA02
Chloroform (HPLC Grade)	Cicarelli	Art. 2906110, CAS N° 67-66-3
Isopropanol (HPLC Grade)	Cicarelli	Art. 2905110, CAS N° 67-63-0
Absolute ethanol	J. T. Baker	CAS °64-17-5
DNase/RNase-free water	PB-L Productos Bio-Lógicos	cat N° IA0602
HCl	Sigma-Aldrich	320331
TAE buffer 50X pH 8,3 tris-acetate EDTA buffer	Merck Millipore	1.06174
UltraPure Agarose	Invitrogen	REF 16500-500

**Experimental models: organisms/strains**		

2-25 weeks-old C3H/HeJ Kcnq4^+/+^ mice from both sexes.	The Jackson Laboratory	RRID: IMSR_JAX:000659
2-25 weeks-old C3H/HeJ Kcnq4^−/−^ mice from both sexes.	Max-Delbrück- Centrum für Molekulare Medizin and Leibniz-Institut für Molekulare Pharmakologie, Berlin, Germany (Prof. Thomas Jentsch)	–

**Others**		

Petri dish 90x15 mm	Henso	REF N1424006
Pestle for 1.5 mL Micro Centrifuge Tube	JBF	CSP00100
1.5 mL Micro Centrifuge Tube RNAse/DNAse free (sterile)	Henso	N 14059
Refrigerated centrifuge	Yingtai Instrument	TGL16E
Vortex 220-230V∼, 50 Hz, 60W.	Scilogex SCI-VS	Cat N° 821200059999
Drybath Model MK-20	BIOTRAZA	Serie: AS-MK20-1750E.
Power Font, Enduro,300V/1V, E0303 Model	Labnet series	N°1203781.
Transilluminator, 9814- series Mini Tables	Cole Parmer	#EW-09814-81
Horizontal Electrophoresis tank BK-HET02	BIOBASE	BK-HET02
Liquid nitrogen	Air Liquide	–
Stereomicroscope Stemi 305 cam EDU Set with K LED illuminators	Zeiss	Serial-No.: 3947019022
Picodrop Pico100, μl Spectrophotometer	Picodrop Ltd.	Serial No: 000383/1
Blue/Orange Loading Dye, 6X	Promega	Catalog Number: G1881

## Materials and equipment

### Recipes of solutions and reagents

**Table:** 10X PBS

Reagent	Final concentration	Amount
Distilled H_2_O (PB-L Productos Bio-Lógicos, cat N° IA0602).		800 mL
KCl (≥99.0 %; Sigma Aldrich, CAS 7447-40-7).	27 mM	2.0 g
NaCl (≥99.0 %; Sigma Aldrich, CAS 7647-14-5).	1370 mM	80 g
Na_2_HPO_4_ (anhydrous; Biopack, CAS [7558-79-4]).	100 mM	14.4 g
KH_2_PO_4_ (anhydrous; Biopack, CAS [7778-77-0]).	18 mM	2.4 g

***Note:*** Adjust to pH 7.4 with HCl (Sigma Aldrich, 320331) and complete to 1000 mL with distilled H_2_O. Then, mix thoroughly and store at 20°C–25°C.

**CRITICAL:** HCl is a hazardous reagent. It is corrosive and can cause severe skin and eye irritation, as well as respiratory harm if inhaled. When handling this acid, use appropriate personal protective equipment (gloves, lab coat, and eye protection) and work in a well-ventilated area or fume hood to avoid exposure.

***Alternatives:*** Use commercially available reagent.

• 1X PBS.
  ○ 100 mL of 10X PBS.
  ○ 900 mL of ultrapure H_2_O (PB-L Productos Bio-Lógicos, N° cat IA0602).

***Note:*** Mix well and store at 4°C.

• 70% Ethanol.
  ○ 700 μL of 100% ethanol (J.T. Baker CAS 64-17-5).
  ○ 300 μL of ultrapure H_2_O (PB-L Productos Bio-Lógicos, cat N° IA0602).

***Note:*** Mix well and store at −20°C.

### Equipment setup

• Centrifuges, rotors, and tube holders should be precooled to 4°C before starting the RNA isolation procedure.
• Dissection tools (scissors, forceps, etc.) should be autoclaved prior to use.
• Work surfaces should be cleaned thoroughly with 70% ethanol before and after dissection.
• Prepare a stereomicroscope for the cochlear dissection.
• Ensure that all reagents are maintained on ice during the entire procedure to preserve RNA integrity.

## Step-by-step method details

### Mouse cochlea isolation

**Timing: 30 min**

At this step, 500 mL 1X PBS should be prepared and kept on ice. All working surfaces must be cleaned with 70% ethanol. Place ice-cold 1X PBS in cell culture dishes immediately before dissection. It is essential that cochlea dissection is performed as quickly as possible and that the isolated cochleae are maintained on ice-cold 1X PBS to preserve RNA integrity. Timing for all subsequent steps is optimized for the isolation of three mice. If additional cochleae are required, timing should be adjusted accordingly.

1. Euthanize and isolate cochleae from mice.
  a. Euthanize mice in CO_2_ chamber or by decapitation, according to institutional and international ethical guidelines.
    ***Note:*** Euthanasia by cervical dislocation is not recommended, as it can cause tissue rupture and blood accumulation, which in turn leads to RNA degradation and reduced yield.
  b. Using No. 5 surgical scissors remove the skin and connective tissues surrounding the skull.
  c. Remove the anterior portion of the head (snout region), then make a midline incision along the skull starting at the foramen magnum and extending rostrally to expose the cranial cavity.
  d. Carefully remove the cranial bone over each hemisphere to expose the brain using N° 5 forceps (Figure 1A).

**Figure 1:**
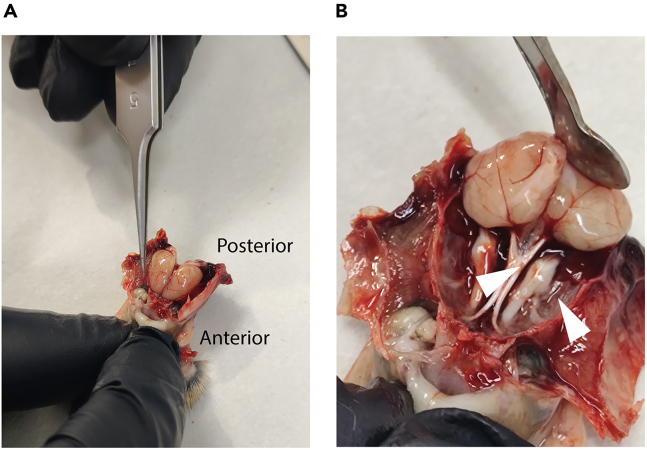
Exposure of the mouse brain and associated nerves (A) Upper view of the mouse brain exposed after careful removal of the skull, depicting the major brain structures *in situ.* (B) Cranial nerves (white arrowheads) are visible beneath the brain, extending toward and connecting with peripheral sensory and motor structures.
  e. Remove the brain using a spatula.
  f. Using fine scissors, carefully sever the nerves connecting the ear to the brainstem (Figure 1B).
  g. Isolate the temporal bone and remove soft tissue.
    i. Position it ventral side up.
    ii. Open the tympanic bulla to expose the middle-ear cavity.
    iii. Rotate the bone laterally to visualize the semicircular canals as landmarks of the bony labyrinth.
    iv. Using micro-scissors, separate the inner ear by cutting along natural bone fissures.
    v. Trim excess bone to outline the auditory bulla, avoiding pressure on the cochlea.
    vi. Using fine forceps, gently release the remaining connective attachments from the lateral side, keeping the auditory bulla intact,
    vii. Lift the inner ear from the temporal bone (For further details, please see Sakamoto et al., 2017^5^).
      **CRITICAL:** Perform this major step as quickly as possible to minimize RNA degradation.
2. Immediately transfer both cochleae into 1X PBS.
  a. Place ice-cold 1X PBS in a 35 mm diameter cell culture dish.
  b. Transfer both inner ears into the dish containing PBS.
  c. Position the cell culture dish under the stereomicroscope.
  d. Using fine-tipped forceps (No. 5, Dumont 11254-20), remove all tissue surrounding the otic capsule.
  e. Open the auditory bulla using iris scissors (No. 5, 14060-09), creating a small fracture along its thinner regions.
    i. Gently widen the opening to expose the cochlea.
    ii. Remove the intact inner ear from the bulla with minimal mechanical disruption.
  f. Using Iris scissors (No. 5, 14060-09), remove the vestibular apparatus (Figure 2A) and isolate the cochlea (Figure 2B).

**Figure 2:**
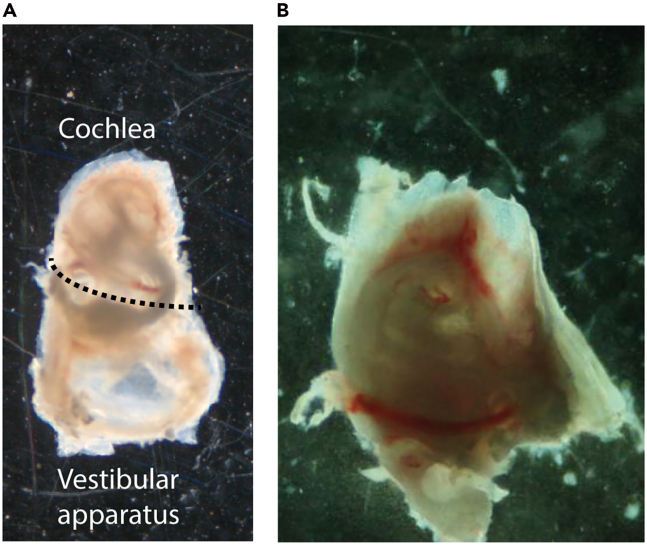
Dissection of the mouse inner ear (A) The cochlea with the attached vestibular apparatus following extraction from the temporal bone. (B) Isolated cochlea after removal of vestibular structures, showing the compact spiral morphology.
3. Freeze the cochleae.
  a. Place both cochleae into a 1.5 mL microcentrifuge tube and immediately snap-freeze them in liquid nitrogen for 10 sec (Figure 3A).

**Figure 3:**
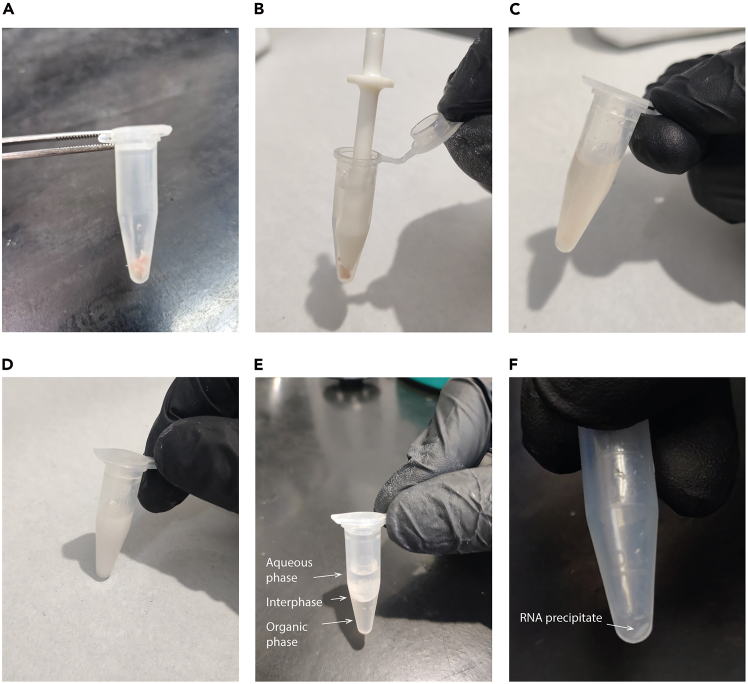
Cochlear RNA extraction procedure (A) Snap freezing of cochlear tissue. Dissected cochleae placed in a microcentrifuge tube immediately after immersion in liquid nitrogen, demonstrating the rapid snap freezing process used to preserve RNA integrity. (B) Cochlear tissue homogenization in Bio-Zol reagent. Cochleae are transferred to a microcentrifuge tube containing Bio-Zol reagent and homogenized thoroughly using a disposable plastic pestle to ensure complete cell lysis. (C) Chloroform addition to the homogenate. Solution obtained after addition of chloroform to the homogenized sample. (D) Mixing after chloroform addition. Milky homogenate observed after vigorous mixing, prior to centrifugation and phase separation. (E) Phase separation during RNA extraction. After centrifugation (step 17 of the protocol), two distinct phases become evident: an upper aqueous phase containing RNA and a lower organic phase retaining DNA and other organic components. (F) RNA pellet. Representative picture of the RNA pellet obtained after isopropanol addition (step 22).
  b. Store the tube at −20°C while dissecting the second mouse.

***Note:*** Replace the ice-cold 1X PBS prior to dissect a new mouse.

**CRITICAL:** For N_2_ handling, wear insulated gloves, safety goggles, and a face shield, to protect against liquid nitrogen and cold gas. Also wear closed-top shoes, long pants, and long-sleeved shirts to avoid liquid nitrogen from spilling onto the skin.

4. Repeat steps 1 and 2 for the second mouse.
  a. Add the third cochlea to the tube containing the cochleae from the first mouse.
  b. Place the other cochlea in a new 1.5 mL microcentrifuge tube.
  c. Immediately snap-freeze both tubes in liquid nitrogen for 10 sec.
  d. Store both tubes at −20°C.
5. Repeat steps 1–4 for the third animal.
  a. Add the last two cochleae to the second tube to pool three cochleae per tube.

***Note:*** This protocol is designed to process three cochleae per tube, to ensure sufficient RNA yield for downstream applications.

### Cochleae homogenization

**Timing: 10 min**

In this step, all the cochleae must be well homogenized and mixed with the Bio-Zol reagent.

**CRITICAL:** Bio-Zol reagent is toxic upon skin contact. Be sure to wear a lab coat, gloves and safety glasses when handling this reagent. If in contact with skin, wash immediately with plenty of neutral soap and water.

***Alternatives:*** Similar results could be obtained with other reagents such as: TransZol (TransGen Biotech, ET101-01), and TRIzol (Invitrogen, 15596026).

6. Immediately add 750 μL of Bio-Zol reagent (corresponding to 250 μL per cochlea) to each tube containing the frozen cochleae.
7. Immediately grind the tubes on ice and homogenize several times using a plastic pestle (JBF) (Figure 3B). Perform several pressing and rotating movements until complete homogenization is achieved.

***Note:*** Ensure that the Bio-Zol reagent reaches the bottom of the tube and no tissue fragments remain visible.

8. Allow the homogenates to rest on ice for 3–5 minutes to let the debris settle. An alternative option could be to perform a spin down with a desktop mini-fuge.
9. Repeat step 7 to further disrupt any remaining sediment.
10. Let the homogenates rest at 20°C–25°C for 5 min.
11. Centrifuge at 12,000 x g for 5 min at 4°C to remove debris.
12. Transfer the supernatants to a fresh 1.5 mL microcentrifuge tubes.

### Aqueous phase separation

**Timing: 20 min**

In this major step, the RNA separates from other molecules such as DNA, proteins and lipids. After chloroform addition and centrifugation, the RNA will be located in the upper aqueous phase, while the proteins and lipids remain in the interphase, and the DNA in the lower organic phase.

13. Add 150 μL of chloroform for every 750 μL of Bio-Zol (Figure 3C) at each tube.

**CRITICAL:** Chloroform is toxic and a potential carcinogen, so it should be handled with extreme caution in a fume hood. Be sure to wear a lab coat, gloves and safety glasses when working with it.

14. Perform a gentle vortex for 15 sec to ensure proper mixing.

***Note:*** The solution should appear milky white (Figure 3D).

15. Incubate the tubes at 20°C–25°C for 3 min.
16. Centrifuge at 12,000 x g for 15 min at 4°C.

***Note:*** After centrifugation, two phases will be visible: a clear upper aqueous phase and a lower organic phase, separated by a white interphase (Figure 3E).

17. Carefully transfer the upper aqueous phases to a fresh RNase-free 1.5 ml microcentrifgue tube.

**CRITICAL:** Do not disturb the interphase in order to avoid protein and lipid contamination. It is preferable to leave a small amount of the aqueous phase rather than risk collecting material from the interphase.

***Note:*** Pool both aqueous phases into a single microcentrifuge tube. Record the final volume in order to calculate the volume of isopropanol required for the next step (approximate final volume is typically 500 μL).

### RNA precipitation

**Timing: 30 min**

In this major step, the RNA is precipitated from the aqueous phase using isopropanol. Precipitation allows the RNA to be concentrated and separated from the remaining soluble components.

18. Add 0.5 volumes of isopropanol to the collected aqueous phase. For example, add 250 μL of isopropanol to 500 μL of aqueous phase.
19. Gently pipette up and down up to three times.
20. Perform a gentle vortex.
21. Incubate the sample for 20 min at −20°C to allow RNA precipitation.
22. Centrifuge the tube at 12,000 x g for 10 min at 4°C.

After centrifugation, a small white or translucent pellet should be visible at the bottom of the tube, representing the precipitated RNA (Figure 3F).

### RNA washing

**Timing: 15 min**

In this step, the RNA pellet will be washed to eliminate contaminants such as residual salts, phenol, and other contaminants. Proper washing is crucial to ensure high RNA purity for downstream applications.

23. Carefully discard the supernatant without disturbing the RNA pellet.
24. Add 500 μL of ice-cold 70% ethanol to the tube.
25. Gently pipette up and down to dislodge and dissolve the pellet.
26. Centrifuge at 12,000 x g for 5 min at 4°C.
27. Carefully discard the supernatant without disturbing the pellet.

***Note:*** Be careful to not disturb the pellet while collecting the supernatant. The pellet may be invisible. Take the supernatant placing the tip in the opposite wall to the precipitate, and pipette up carefully. When the precipitate region is reached (Figure 3E), lay down the tube and carefully remove the rest of supernatant.

28. Air-dry the tube less than 10 min at 20°C–25°C under laminar flow. Ensure that no ethanol residues remain.

### RNA elution

**Timing: 5 min**

In this step, the purified RNA is resuspended in RNase-free water for immediate use or long-term storage. Proper elution ensures complete RNA recovery and stability.

29. Add 25 μL of RNase-free water preheated to 55°C on the pellet.
30. Pipette up and down several times to facilitate pellet dissolution.
31. Incubate the tube for 5–10 min at 55°C.
32. Separate 5 μL for RNA evaluation.
33. Immediately store RNA samples at −80° until further use.

### RNA measurement

**Timing: 5 min**

**Figure 4:**
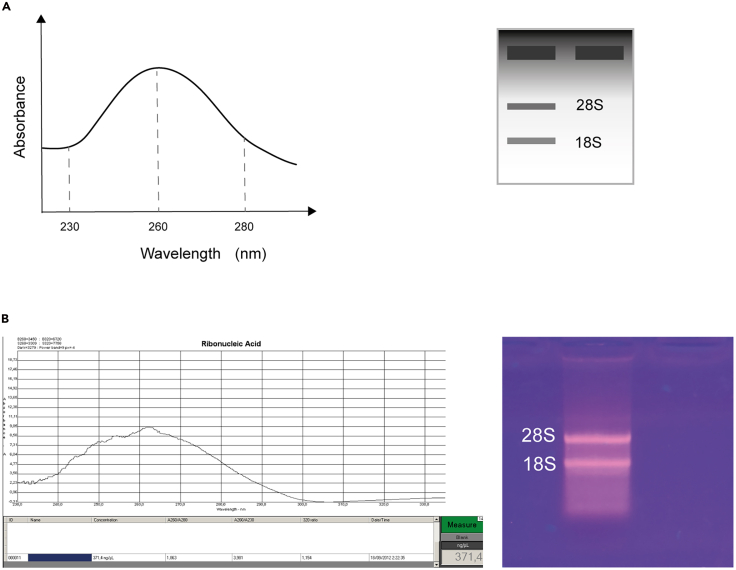
RNA quality assessment from mouse cochlea samples (A) *Left*. Schematic expected absorbance spectrum of RNA solution depicting the 230, 230, and 280 absorbances. *Right*. Schematic agarose gel electrophoresis depicting expected the bands obtained from a RNA sample. (B) *Left*. Absorbance spectrum of total RNA extracted from mouse cochlea using the present protocol, showing characteristic peaks at 260 and 280 nm used to evaluate purity, obtained with the Picodrop Pico100 and the software Picodrop application v.4.0.4.0. *Right*. Agarose gel electrophoresis of total RNA extracted from mouse cochlea using the present protocol, displaying two clears ribosomal RNA bands (28S and 18S), indicative of high RNA integrity and minimal degradation.

In this final step, the quality and quantity of the obtained RNA are evaluated (Figure 4A). Electrophoresis on an agarose gel is used to assess RNA integrity. The absorbance values of the RNA solution allow calculation of the RNA concentration and assessment of potential contaminates.

34. Pipette 2 μL of the RNA solution to evaluate RNA quantity.
35. Using a UV spectrophotometer, measure absorbances at 230, 260, and 280 nm. Some instruments directly display the concentration (Figure 4B, left). If it is not the case, using absorbances calculate the concentration applying the following formula:
  a. Concentration(μg/mL)=(A260absorbance)×40(μg/mL)(RNAconversionfactor)×dilutionfactor.
  b. Using the absorbances at 280 and 230, calculate the 260/280 and 260/230 ratios, indicatives of protein and solvent contamination, respectively.
    ***Note:*** Any UV spectrophotometer can be used for this purpose. In this case we used the Picodrop Pico100 (Picodrop Ltd., Sonntek, USA).
36. To verify RNA integrity, perform an agarose gel electrophoresis.
  a. Prepare a 1% agarose gel and use freshly prepared 1X TAE buffer.
  b. Prepare the loading sample by mixing 2 μL of RNA solution with 1 μL of 6X loading dye (commercial or homemade) and bringing the final volume to 6 μL with nuclease-free water.
  c. Load the entire 6 μL onto the agarose gel and run the gel for 10 min at 80 V.
  d. Visualize the gel using a UV-transilluminator to identify RNA bands (Figure 4B, right).

**CRITICAL:** When visualizing the agarose gel, wear gloves and eye protection. Avoid exposure to UV radiation.

## Expected outcomes

Following completion of this protocol, high-quality total RNA should be obtained from mouse cochleae. The resulting RNA solution should be homogeneous, transparent, and free of precipitates.

Spectrophotometric analysis is expected to show a maximum absorbance peak at 260 nm, corresponding to nucleic acid content (Figure 4A, left), with an A260/A280 ratio between 1.8 and 2.1, indicating high purity. Typical yields range from 150 to 500 ng/μL, depending on the number and condition of the cochleae processed. Furthermore, when assessed by agarose gel electrophoresis, intact RNA will display two well-defined ribosomal bands (28S and 18S), confirming its structural integrity and absence of degradation (Figure 4A, right). Our research group has successfully applied this protocol for the extraction and analysis of cochlear RNA in both WT and *Kcnq4*^−/−^ mouse models, obtaining reproducible, high-quality results suitable for reverse transcription, qPCR, and gene expression studies.^1^

## Limitations

This protocol was developed to provide a cost-effective and reliable alternative to commercial RNA extraction kits, while maintaining comparable yield and purity. However, several limitations should be considered when applying it to cochlear tissue.

The first limitation, and probably the most important, concerns the number of animals required. As described, the protocol is optimized to process six cochleae (from three mice), pooling three cochleae per tube. Fewer samples may result in reduced RNA yield, potentially affecting downstream applications such as quantitative PCR or RNA sequencing. The second limitation relates to processing time. Because RNA is highly susceptible to degradation, all dissection and extraction steps must be performed quickly and efficiently, keeping tissues and reagents constantly on ice to preserve RNA integrity. Finally, the third limitation involves the temperature and handling of reagents. All solutions should be precooled, and samples must be processed on ice to minimize RNase activity and prevent RNA structural damage or degradation.

Despite these constraints, this protocol provides a reproducible, accessible, and robust method for isolating high-quality total RNA from the mouse cochlea, suitable for a wide range of downstream molecular analyses.

## Troubleshooting

Below are common issues that may arise during the RNA isolation process, along with potential causes and recommended solutions.

### Problem 1

Low RNA yield.

### Potential solution

During the initial steps (steps 12), ensure to collect the entire volume when separating the supernatant from the debris, in order to minimize sample loss.

Ensure thorough homogenization of cochlear tissue in Bio-Zol reagent, allowing the reagent to reach all tissue fragments (steps 6–12).

Properly elute the RNA precipitate with H_2_O to ensure complete resuspension (steps 29–31).

When washing with 70% ethanol, carefully lift and recover the RNA pellet to maximize yield and prevent material loss (steps 24–26).

### Problem 2

Contamination from the interphase (step 17).

### Potential solution

If the interphase is accidentally collected, return aqueous phase together with the interphase to the same tube and repeat the centrifugation step to improve phase separation and ensure cleaner recovery of the aqueous layer.

### Problem 3

Presence of ethanol residues (step 28).

### Potential solution

Slightly increase the drying time or place the sample at 55°C for less than 5 minutes to ensure complete evaporation of ethanol without compromising RNA integrity.

### Problem 4

Appearance of a white precipitate during RNA precipitation (step 22).

### Potential solution

Ideally, the sample should be discarded and RNA extraction should be restarted using fresh tissue. If that is not possible, separate the supernatant from the pellet after centrifugation in step 22 (Centrifuge at 12,000 × g for 10 min at 4°C).

## Resource availability

### Lead contact

Further information and requests for resources and reagents should be directed to and will be fulfilled by the lead contact, Dr. Leonardo Dionisio (ldionisio@inibibb-conicet.gob.ar).

### Technical contact

Further information for technical concerns should be directed to the technical contact, PhD student Ezequiel Rías (erias@inibibb-conicet.gob.ar).

### Materials availability

This study did not generate new unique reagents.

### Data and code availability

• All relevant data are available within the article or from the lead contact upon reasonable request.
• This paper does not report original code.

## Acknowledgments

Financial support for G.S. was provided by the National Agency for the Promotion of Research Technological Development and Innovation and by the National University of the South. Financial support for L.D. was provided by the National Agency for the Promotion of Research Technological Development and Innovation and by the National University of the South. E.R. has a doctoral fellowship from the Argentina National Research Council (CONICET).

## Author contributions

Conceptualization, E.R. and L.D.; methodology, E.R. and L.D.; validation, E.R. and L.D.; formal analysis, E.R. and L.D.; investigation, E.R. and L.D.; data curation, E.R. and L.D.; writing – original draft, E.R. and L.D.; writing – review and editing, E.R., L.D., and G.S.; visualization, E.R. and L.D.; supervision, L.D.; project administration, L.D. and G.S.; funding acquisition, L.D. and G.S.

## Declaration of interests

The authors declare no competing interests.
